# Robust facial expression recognition system in higher poses

**DOI:** 10.1186/s42492-022-00109-0

**Published:** 2022-05-16

**Authors:** Ebenezer Owusu, Justice Kwame Appati, Percy Okae

**Affiliations:** 1 Department of Computer Science, University of Ghana, P. O. Box LG 163, Accra, Ghana; 2 Department of Computer Engineering, University of Ghana, P. O. Box LG 77, Accra, Ghana

**Keywords:** Facial expressions, Three-dimensional head pose, Ellipsoidal model, Gabor filters, Ada-AdaSVM

## Abstract

Facial expression recognition (FER) has numerous applications in computer security, neuroscience, psychology, and engineering. Owing to its non-intrusiveness, it is considered a useful technology for combating crime. However, FER is plagued with several challenges, the most serious of which is its poor prediction accuracy in severe head poses. The aim of this study, therefore, is to improve the recognition accuracy in severe head poses by proposing a robust 3D head-tracking algorithm based on an ellipsoidal model, advanced ensemble of AdaBoost, and saturated vector machine (SVM). The FER features are tracked from one frame to the next using the ellipsoidal tracking model, and the visible expressive facial key points are extracted using Gabor filters. The ensemble algorithm (Ada-AdaSVM) is then used for feature selection and classification. The proposed technique is evaluated using the Bosphorus, BU-3DFE, MMI, CK + , and BP4D-Spontaneous facial expression databases. The overall performance is outstanding.

## Introduction

### Applications

Facial expression recognition (FER) is the automatic detection of the emotional state of a human face using computer-based technology. The field of study is currently a hotspot of research because it has increasing applications in several domains, such as psychology, sociology, health science, transportation, gaming, communication, security, and business. According to Panksepp [1], facial expressions and emotions guide the lives of people in a variety of ways, and emotions are key aspects that enlighten us in how we should act, from elementary processes to the most intricate acts [2, 3].

The sporadic advancements in the use of facial expressions in neuropsychiatric complications have shown more positive results [4], and current studies are focusing on human behavior and the detection of mental illnesses [5, 6].

FER can also affect data collection in specific research projects. For example, Shergill et al. [7] proposed an intelligent assistant FER framework that could be implemented in e-commerce to determine the product preferences of customers. The system captures the facial data as they browse the e-shop for products to acquire. Based on the facial expression, the systems can automatically suggest more products of possible interest.

Certain physiological features of people have been discovered to be useful as intelligent data in the search for criminals [8, 9]. This theory is based on the tendency for someone with ego to commit a high-profile crime, such as terrorism, exhibits specific emotions such as anger and fear. Consequently, the accurate recognition of these expressions could lead to further security measures in apprehending criminals.

FER can also be valuable during the testing phase of video games. Target groups are frequently invited to play a game for a set amount of time, and their behaviors and emotions are observed as they play. Game developers may acquire more insights and valuable deductions about the emotions recorded during gameplay using FER technology, and incorporate the feedback into production.

### Technical issues on the use of two-dimensional facial data

Two-dimensional (2D) FER systems are extremely sensitive to head orientation. Therefore, to achieve good results, the subject must be constantly in a fronto-parallel orientation. The problem resulting from this is that the throughput of most site-access systems is significantly reduced. This implies that subjects are frequently required to perform several verifications to attain an ideal facial orientation. Consequently, surveillance systems operate on luck, hoping the subject faces the camera.

Another problem that arises from the use of 2D technology is the illumination conditions of the surrounding environment. If the subject is in a setting with varying lighting conditions, FER reduces in accuracy because the FER processes are sensitive to the direction of lighting and the ensuing shading pattern. Consequently, cast shadows may obstruct recognition by concealing informative features.

Three-dimensional (3D) FER systems have a higher detection rate than 2D systems because of their higher intensity modality, and they also have more object description geometry information [10, 11]. This demonstrates the importance of pushing FER into higher face orientations to improve its realism and practicality.

### Related work

The primary focus of this study is to improve FER accuracy in higher facial orientations.

Yadav and Singha [12] adopted the Viola-Jones descriptor [13] to detect faces and used a combination of local binary patterns (LBP) and the histogram of gradients (HOG) as a feature extraction tool. Subsequently, traditional SVM with the k-means method was employed as a training algorithm. LPB feature extraction techniques, such as Gabor, are orientation-selective, and thus, highly robust in tracking key facial features. However, the Viola-Jones descriptor is computationally demanding and has a low detection accuracy. Furthermore, the conventional SVM described in the study is slow to classify. Consequently, the overall architecture used in the study was computationally expensive. Yao et al. [14] proposed a linear SVM method that used AUs to recognize seven facial expression prototypes in the CK database. The Viola-Jones descriptor was used as the face-detection technique again. Although the goal of the study was to minimize computational complexity and enhance recognition accuracy, the resulting average recognition accuracy of 94.07% for females and 90.77% for males was too low for a viable implementation. Ashir et al. [15] also proposed an SVM-based multiclass classification for detecting seven facial expressions across four prominent databases. The Nyquist–Shannon sampling method [16] was used to compress the extracted facial feature samples. Although the sampling method reduces data loss, it is prone to aliasing issues, particularly when the bandwidth is extremely large. The Nyquist-Shannon sampling technique is difficult to deploy because it assumes the sampled signal is completely band-restricted. In real-world applications, this is a concern because no actual signal is genuinely and completely band-restricted. The compressing sampling [17] paradigm could have been a better option because it is less restrictive. Perez-Gomez et al. [18] recently proposed a 2D–3D FER system that used principal component analysis (PCA) and a genetic algorithm for feature selection, and a k-nearest neighbor (KNN)-multiclass SVM for learning. In this study, the synthetic minority oversampling technique (SMOTE) [19] was used to balance the instances. However, SMOTE creates an equal number of synthetic samples for each minority data sample and relies on the hypothesis performance to update the distribution function. The adaptive synthetic (ADASYN) [20] method tends to generate more synthetic data for minority class samples that are harder to learn than with SMOTE, which is easy to learn. In addition, PCA uses observations from all the extracted features in the projection to the subspace and only considers linear relationships, ignoring the input multivariate structures. Compared to other recent studies, the findings of this study were not positive.

Li et al. [21] proposed a robust 3D local coordinate technique for extracting pose-invariant facial features at key points. The descriptor in this method is a multi-task sparse representation fine-grained matching algorithm. The method was evaluated using the Bosphorus datasets, and an average recognition accuracy of 98.9% was obtained. The success of this study is largely owed to the accurate tracking of 3D key points. This recent study is a primary driving force behind our proposed study.

The following are the significant contributions of this work: (1) A robust head-tracking algorithm that tracks facial features from one frame to the next, accounting for more features in the overall prediction process; (2) A unique ensemble approach that employs AdaBoost for feature selection, and a combination of AdaBoost and SVM for classification. AdaBoost is extremely fast, whereas SVM is extremely accurate. Consequently, the proposed technique becomes extremely fast while also improving the recognition accuracy.

The remainder of this paper is organized as follows. Methods section delves into the proposed strategy. Results and discussion section discusses the findings, debates, and analyses. Finally, Conclusions section concludes the study.

## Methods

We robustly tracked the facial features from one frame to the next using 3D facial data. With 3D data, information, such as the size and shape of an object, can be correctly estimated in each frame without prior assumptions.

The first priority is to detect the focal points in each frame. The next step is to search for matching features or objects across all frames. This method addresses the changing behavior of a moving object and the preceding annotations of the scene. In this approach, the location of an object is projected by iteratively updating the object position from previous frames [22, 23].

### Architectural framework

**Fig. 1:**
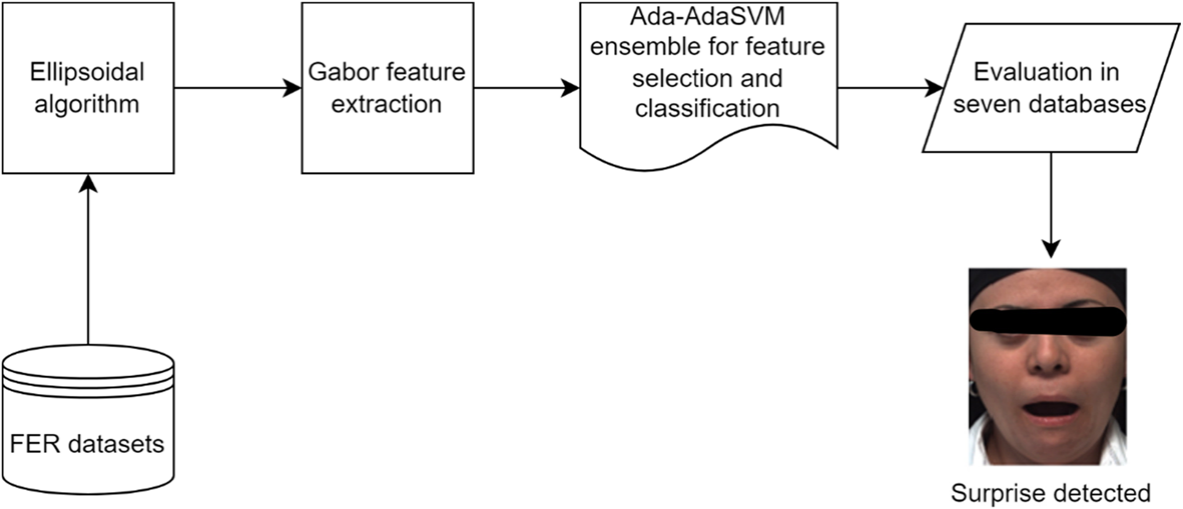
Architectural framework of this study

Figure 1 presents the framework of this study.

This procedure uploads images and robustly tracks the features across frames using the proposed ellipsoidal model. Subsequently, the Gabor feature-extraction approach was used. Feature points extraction section explains the reason for using Gabor features in this study. Feature selection and classification were executed using the Ada-AdaSVM.

### Ellipsoidal feature tracking method

**Fig. 2:**
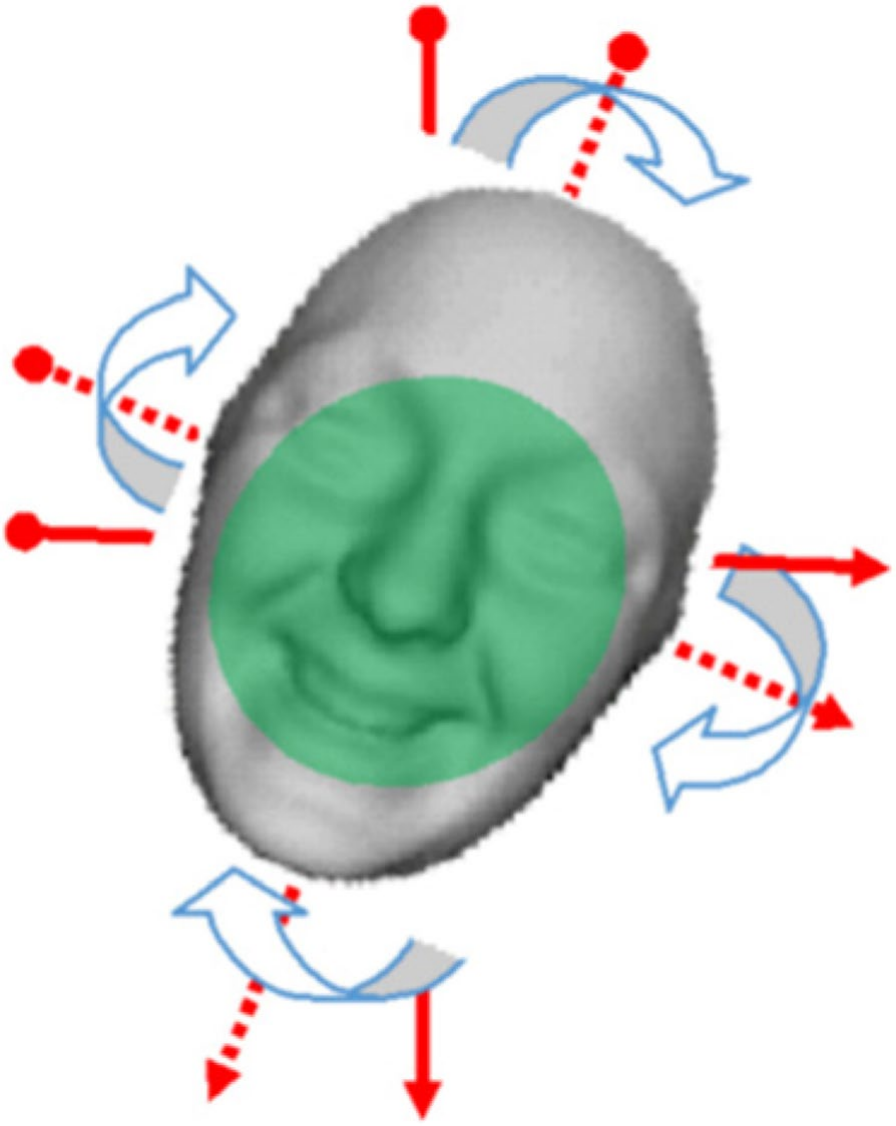
Tracking of 3D feature points from one frame to another

Accurate tracking of a human face from the forehead, to the left cheek, to the chin, to the right cheek, and back to the same spot on the forehead where the tracking began unmistakably demonstrates that the human face is best shaped like an ellipse. Thus, considering the 3D facial representation in Fig. 2 with *N* feature points tracked across frames, we denote:

1 $$\alpha (t) = \left\{ {f_{j} (t)\left| {1 \le j \le N} \right.} \right\}$$

where *N* represents the most relevant feature points. In this study, we assumed *N* to be 24. In addition, let ${f}_{j}(t)\in \alpha (t)$ denote a facial feature. As the features move from one frame to the next at time *t* + 1, the position of feature ${f}_{j}(t)$ becomes ${f}_{j}(t+1)$. Therefore, ${f}_{j}(t+1)\in \alpha (t+1)$. Assuming that ${Y}_{j}$ is the position of ${\alpha }_{j}$ on the 3D facial model and ${\alpha }_{j,p}[\varnothing \left(t+1\right)]$ represents its back projection on the image plane, the 3D facial orientation at *t* + 1 is the vector $\varnothing \left(t+1\right)$ that minimizes ${\sum }_{j=1}^{N}{S}_{j}^{2}$, where:

2 $$S_{j} [\phi (t + 1)] = ||\alpha_{j,p} [\phi (t + 1)] - \alpha_{j} (t + 1)||$$

This is a multi-view system based on the assumption that cameras are positioned around the subject to capture various rotation movements. Consequently, the facial image can be captured with a high degree of precision in any orientation. We extracted the features in the same manner as for 2D images. The right and left eyes, lips, and muscles around the cheeks are important parts of the face to consider. Slight disruptions primarily and severely distort the muscles in these places. The Gabor technique is then used to extract the features of the captured face.

**Fig. 3:**
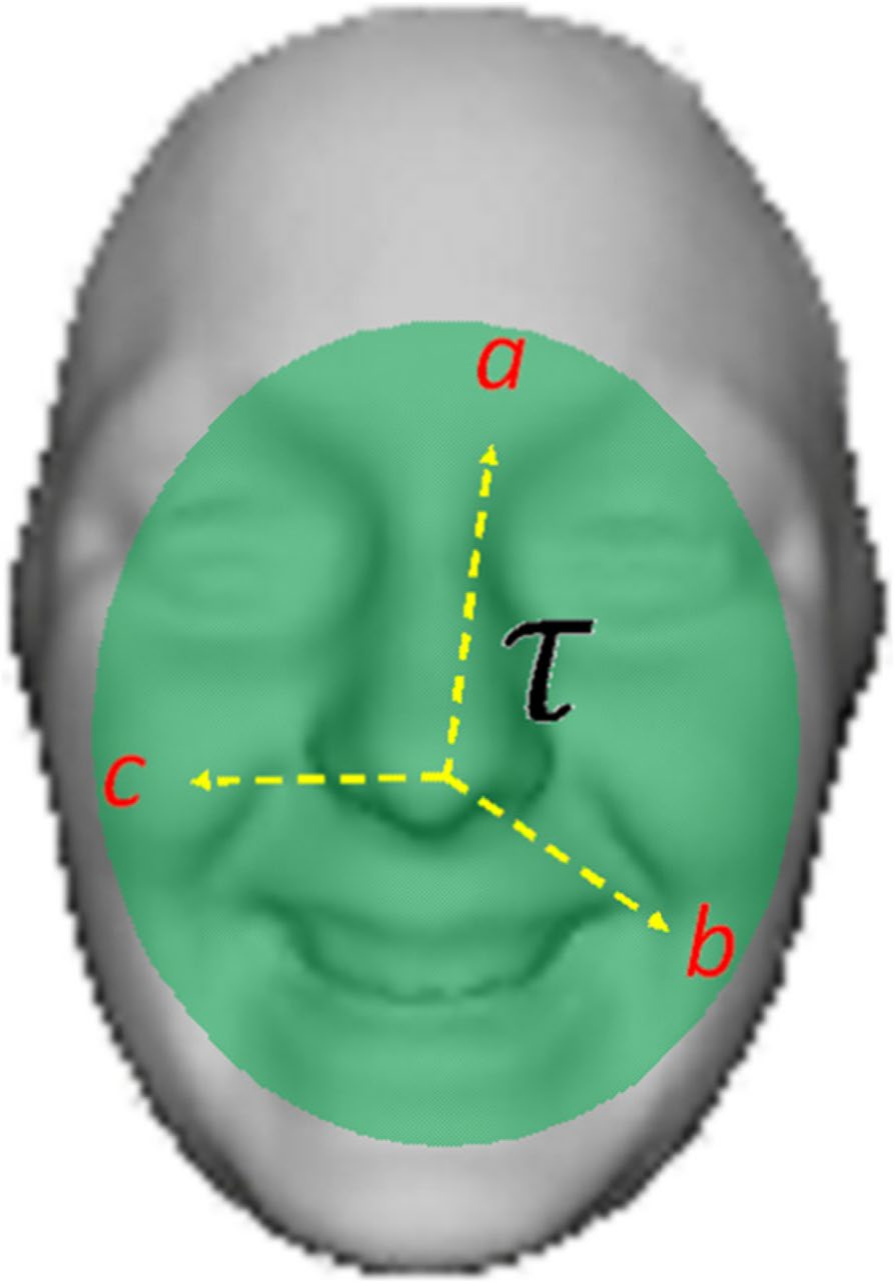
Ellipsoidal face model

The algorithm models a procedure that chooses a set of features and robustly tracks them from one frame to the next while discarding all other features that are no longer required for tracking. The ellipsoidal 3D face was modelled, as shown in Fig. 3.

Adopting homogeneous coordinates for an ellipsoid of the semi-axis, *a*, *b*, and *c*, states that a point ${X}_{0}=\left({x}_{0}, {y}_{0}, {z}_{0}, 1\right)$ belongs to the surface of the ellipsoid if ${X}_{0}^{T}{E}_{0}{X}_{0}=0$.

3 $$E_{0} = \left[ {\begin{array}{*{20}c} {b^{2} c^{2} } & 0 & 0 & 0 \\ 0 & {a^{2} c^{2} } & 0 & 0 \\ 0 & 0 & {a^{2} b^{2} } & 0 \\ 0 & 0 & 0 & { - a^{2} b^{2} c^{2} } \\ \end{array} } \right]$$

**Fig. 4:**
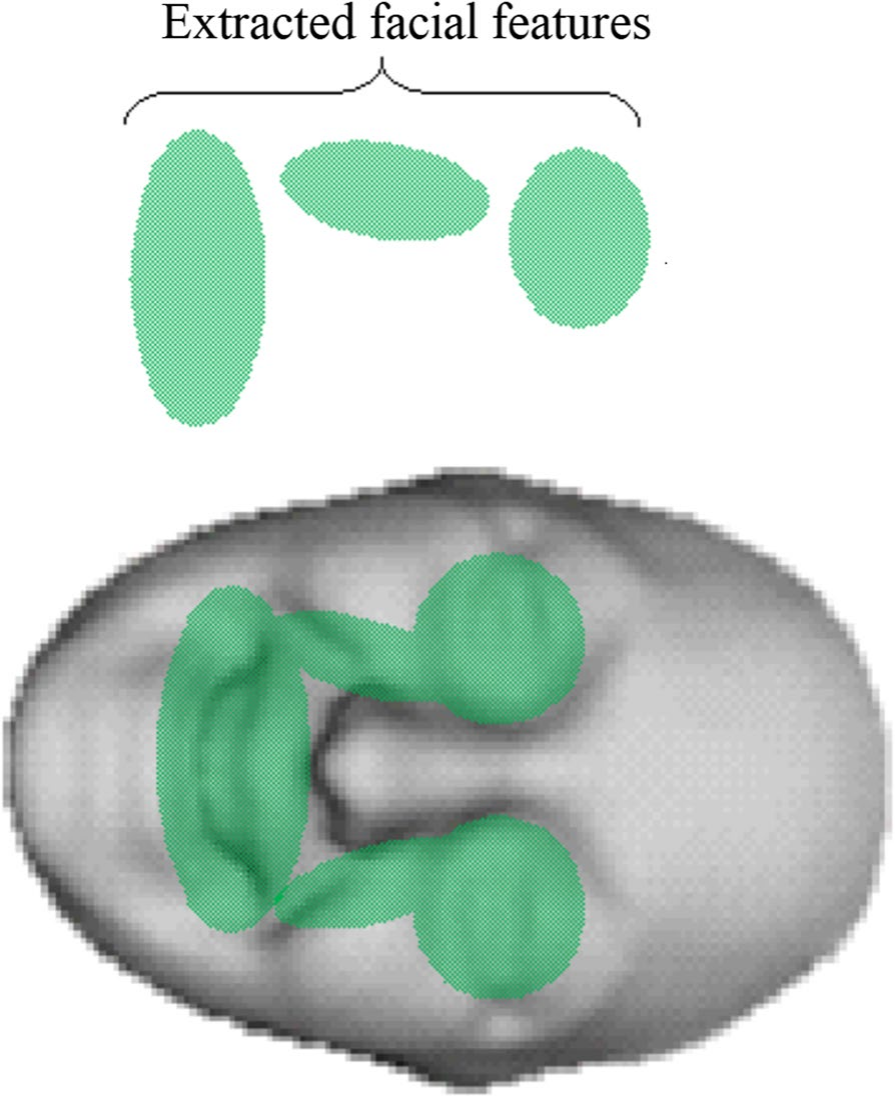
Model of feature extraction points in 3D

The algorithm tracks the facial features that are more noticeable by slight deformation from one frame to the next using the brightness change constraint [24]. These muscles are usually near the eyes, mouth, cheeks, and edges, as shown in Fig. 4 and contour τ in Fig. 3.

Given that pixel (*x*, *y*) with luminance $I{\left(x,y\right)}^{T}$ moves from position (*x*, *y*)^T^ at frame *t* to position ${\left(x+u, y+v\right)}^{T}$ at frame *t* + 1 in high frame rates. In this instance, we can deduce that

4 $$I(x + u,y + v,t + 1) = I(x,y,t)$$

By applying Taylor’s series, and considering *I*_*x*_ and *I*_*y*_ as gradients and that *I*_*t*_ is a temporal deviation of the image, we can infer that

5 $$[I_{x} (x,y,t)I_{y} (x,y,t)]\left( {\begin{array}{*{20}c} u \\ v \\ \end{array} } \right) + I_{t} (x,y,t) = 0$$

If a whole window ${\omega }_{k}$ is considered instead of a single pixel, we deduce that

6 $$J(u,v) = \left[ {\sum\nolimits_{{\omega_{k} }} {I_{x} \left( {x,y,t} \right)\sum\nolimits_{{\omega_{k} }} {I_{y} \left( {x,y,t} \right)} } } \right]\left( {\begin{array}{*{20}c} {u_{k} } \\ {v_{k} } \\ \end{array} } \right) + \sum\nolimits_{{\omega_{k} }} I_{t} \left( {x,y,t} \right) = 0$$

The solution of Eq. (6) is an optimization problem. By introducing the cost function, it follows that

7 $$J(u,v) = \left\{ {\left[ {\sum\nolimits_{{\omega_{k} }} {I_{x} \left( {x,y,t} \right)\sum\nolimits_{{\omega_{k} }} {I_{y} \left( {x,y,t} \right)} } } \right]\left( {\begin{array}{*{20}c} {u_{k} } \\ {v_{k} } \\ \end{array} } \right) + \sum\nolimits_{{\omega_{k} }} I_{t} \left( {x,y,t} \right)} \right\}^{2}$$

The optimal displacement vector that determines the new position of face ${\omega }_{k}$ is given by:

8 $$\left( {\begin{array}{*{20}c} {u_{k} } \\ {v_{k} } \\ \end{array} } \right) = \arg \underbrace {\min }_{{\left( {\begin{array}{*{20}c} u \\ v \\ \end{array} } \right) \in {\mathbb{R}}^{2} }}J(u,v)$$

where, (*u*_*k*_, *v*_*k*_) represents the image at a new position. By computing the derivative of *J* with respect to *u* and *v* and equating them to zero, we obtain:

9 $$C_{k} \left( {\begin{array}{*{20}c} {u_{k} } \\ {v_{k} } \\ \end{array} } \right) + D_{k} = 0$$

where $${C}_{k}=\left(\begin{array}{cc}\sum_{{\omega }_{k}}{I}_{x}^{2}& \sum_{{\omega }_{k}}{I}_{x}{I}_{y}\\ \sum_{{\omega }_{k}}{I}_{x}{I}_{y}& \sum_{{\omega }_{k}}{I}_{y}^{2}\end{array}\right)$$, and $${D}_{k}=\left(\begin{array}{c}\sum_{{\omega }_{k}}{I}_{x}{I}_{t}\\ \sum_{{\omega }_{k}}{I}_{x}{I}_{t}\end{array}\right)$$. Assuming that $$I: \left[1, m\right]\times \left[1, n\right]\subseteq$$$${\mathbb{N}}^{2}\to \left[0, 1\right]$$ is the matrix of the 3D face, then the *j*^*th*^ level of the pyramid description of the face image is expressed by the recursion:

10 $$I{}^{j}(x,y) = \left\{ {\begin{array}{*{20}c} { \, I(x,y) \,,j = 0} \\ \begin{gathered} \hfill \\ \frac{1}{4}I^{j - 1} (2x,2y) + \hfill \\ \frac{1}{8}[I{}^{j - 1}(2x - 1,2y) + I^{j - 1} (2x + 1,2y) + \hfill \\ I^{j - 1} (2x,2y - 1) + I^{j - 1} (2x,2y + 1)] + \hfill \\ \frac{1}{16}[I^{j - 1} (2x - 1,2y - 1) + I^{j - 1} (2x + 1,2y + 1) + \hfill \\ I^{j - 1} (2x + 1,2y - 1) + I^{j - 1} (2x - 1,2y + 1)] \hfill \\ \end{gathered} \\ \end{array} } \right., j \ne 0$$

The displacement vector in Eq. (9) can also be rewritten as:

11 $$\left( {\begin{array}{*{20}c} {u_{k} } \\ {v_{k} } \\ \end{array} } \right) = - C_{k}^{ - 1} D_{k}$$

The displacement vector in Eq. (10) is computed at the deepest pyramid level ${j}_{max}$ (in the Newton–Raphson fashion), and the result of the computation is propagated to the upper level ${j}_{max}-1$ by the expression:

12 $$\left( {\begin{array}{*{20}c} {u_{k}^{j - 1} } \\ {v_{k}^{j - 1} } \\ \end{array} } \right) = 2\left( {\begin{array}{*{20}c} {u_{k}^{j} } \\ {v_{k}^{j} } \\ \end{array} } \right)$$

Equation (12) was used as the initial estimate for the evaluation of the displacement vector of the 3D face. The final displacement vector is given by the expression

13 $$\left( {\begin{array}{*{20}c} {u_{k} } \\ {v_{k} } \\ \end{array} } \right) = \sum\limits_{j = 0}^{{j_{\max } }} {2^{j} \left( {\begin{array}{*{20}c} {u_{k}^{j} } \\ {v_{k}^{j} } \\ \end{array} } \right)}$$

The visible features of the face can be extracted from any location on the face, similar to any other 2D dimensional face. The extracted features are candidates for predicting the overall expression of the face. The Gabor extraction technique is critical for extracting the maximum amount of information required for the classifier.

### Feature points extraction

**Fig. 5:**
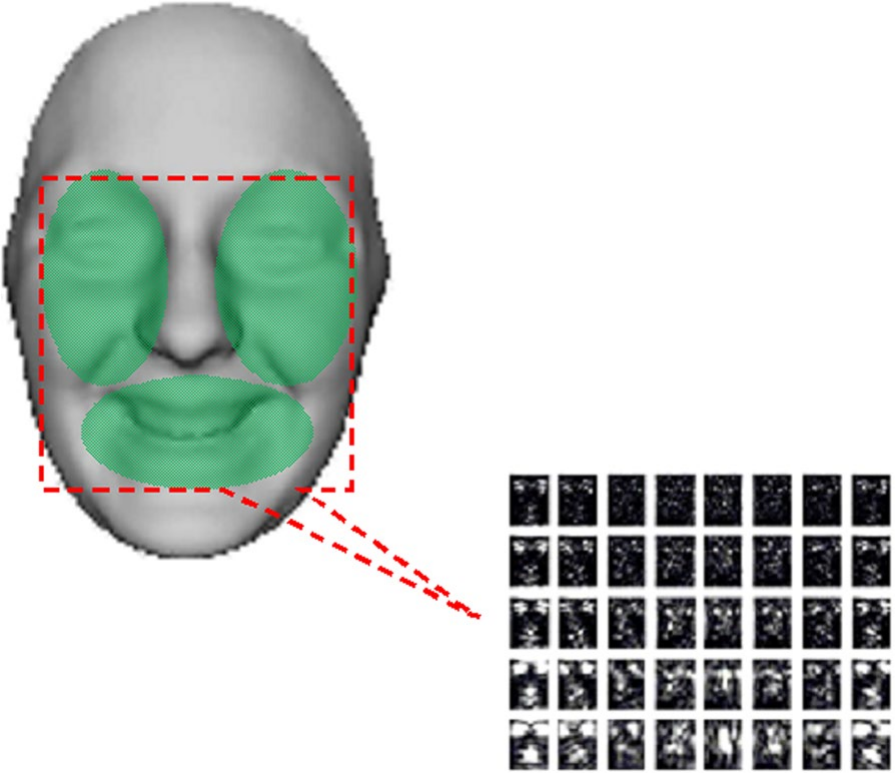
Gabor magnitude response of the expressive face image: sample image (left), magnitude response image of the whole Gabor filter bank of 40 Gabor filters (right)

The 2D Gabor filters are spatial sinusoids localized by the Gaussian window, and because they are orientation-, localization-, and frequency-selective, they are useful in this study. Demonstrate images using Gabor wavelets provides flexibility because the details about their spatial relations are preserved in the process. The general form of the Gabor function is given by:

14 $$G(x,y,\theta ,u,\sigma ) = \frac{1}{{2\pi \sigma^{2} }}\exp \left\{ { - \frac{{x^{2} + y^{2} }}{{2\sigma^{2} }}} \right\}\exp \left[ {2\pi i(R_{1} + R_{2} )} \right]$$

where ${R}_{1}=uxcos\theta$ and ${R}_{2}=uysin\theta$, *u* is the spatial frequency of the band pass, *θ* is the spatial orientation, $\sigma$ is the standard deviation that the 2D Gaussian envelops, and (*x*, *y*) is the position of the light impulse in the visual field. To allow for more robustness in illumination, we set the filter to zero direct current. The Gabor wavelet is then given by:

15 $$\tilde{G}(x,y,\theta ,u,\sigma ) \simeq G(i,j,\theta ,u,\sigma ) = \frac{1}{q}\left[ {\sum\limits_{i = - n}^{n} {\sum\limits_{j = - n}^{n} {G(} } x,y,\theta ,u,\sigma )} \right]$$

where $\left(x,y, \theta ,u,\sigma \right)$ are parameters with (*i, j*) being the new position of the 2D input point, $\theta$ is the scale, *u* is the orientation of the Gabor kernel, $\sigma$ is the standard deviation of the Gaussian window in the kernel, *n* is the maximum size of the face peak, and *q* is the size of the filter given by $q={\left(2n+1\right)}^{2}$. In this study, we used 8 orientations given by $\left\{ {0,\frac{\pi }{8},\frac{\pi }{4},\frac{3\pi }{8},\frac{\pi }{2},\frac{5\pi }{8},\frac{3\pi }{4},\frac{7\pi }{8}} \right\}$ and 5 scales given by $\left\{ {4,4\sqrt 2 ,8,8\sqrt 2 ,16} \right\}$. The sample points of the filtered image are coded into two bits $\left({x}_{1}, { x}_{2}\right)$ such that:

16 $$G_{1} = \left\{ {\begin{array}{*{20}c} {x_{1} = 1,if\left\{ {\Re [\tilde{G}(x,y,\theta ,u,\sigma )] * I} \right\} \ge 0} \\ {x_{1} = 0,if\left\{ {\Re [\tilde{G}(x,y,\theta ,u,\sigma )] * I} \right\} < 0} \\ \end{array} } \right.$$

17 $$G_{2} = \left\{ {\begin{array}{*{20}c} {x_{2} = 1,if\left\{ {\Im [\tilde{G}(x,y,\theta ,u,\sigma )] * I} \right\} \ge 0} \\ {x_{2} = 0,if\left\{ {\Im [\tilde{G}(x,y,\theta ,u,\sigma )] * I} \right\} < 0} \\ \end{array} } \right.$$

where *I* is a sub-image of the expressional face; $\mathfrak{R}$ and $\mathfrak{I}$ are the real and imaginary parts of each Gabor kernel, respectively; and the star (*) is the convolution operator. The final magnitude response, representing the feature vectors, was computed by determining the square root of the sum of the squares of *G*_*1*_ and *G*_*2*_. Figure 5 shows the magnitude response of a template image.

### Classification using Ada-AdaSVM

For this optimization problem, an SVM with a radial basis function kernel was used as a weak classifier. This weak SVM classifier was trained to produce the optimum Gaussian value for the scale parameter $\delta$ and regularization parameter $\partial .$ Typically, the best parameters are $\left\{ {^{\prime}\partial ^{\prime}:1.0,{ ^{\prime}}\delta {^{\prime}: 0}{\text{.1}}} \right\}$. The feature selection hypothesis is then computed from the expression $sgn\left[{\sum }_{t-1}^{T}{\omega }_{t}{h}_{t}^{1}\left({\varphi }_{t}^{1}\right)\right]$, where *T* is the final iteration, ${h}_{t}^{1}$ is the hypothesis with the most discriminating information, and ${\omega }_{t}$ is weights that weigh ${h}_{t}^{1}$ based on its classification performance. The learning process formulated in our recent study [25] is as follows:

Step 1: Input the training sets, $[\left({y}_{1}, {x}_{1}\right), \left({y}_{2}, {x}_{2}\right),\dots , \left({y}_{N}, {x}_{N}\right)]$, $N=a+b$; where datasets *a* and *b* comprise ${y}_{i}=+1$ and ${y}_{i}=-1$ datasets, respectively. Initially, $\delta = {\delta }_{ini}, { \delta }_{min}, {\delta }_{step}$. The scale parameter $\delta$, *x,* and *y* are the feature vectors selected by the AdaBoost algorithm.

Step 2: Initialize the training set weights, ${w}_{i}^{(1)}=1\left/ 2a\right., \forall \left({y}_{i}=+1\right)$ and ${w}_{i}^{(1)}=1\left/ 2a\right., \forall \left({y}_{i}=-1\right)$.

Do while $$\delta >{\delta }_{min}$$

Step 3: Apply the RBFSVM kernel to train the weighted training datasets by applying the leave-one-subject-out cross validation (LOSOCV) approach and compute the training error for the weak classifier ${h}_{t}$ as

18 $$\xi_{t} = \sum\nolimits_{i = 1}^{N} {w_{i}^{t} } ,y_{i} \ne h_{t} (x_{i} )$$

Step 4: At ${\xi }_{t}=1\left/ 2\right.$, reduce $\delta$ by a factor of ${\delta }_{step}$ and then jump to Step 1.

Step 5: Place the weight of the constituent classifier ${h}_{t}$ such that

19 $$h_{t} :\alpha_{t} = \ln \left[ {\frac{1}{{\xi_{t} }} - 1} \right]^{\frac{1}{2}}$$

Step 6: Update the weights by computing:

20 $$w_{i}^{t + 1} = \frac{{w_{i}^{t} \exp \left\{ { - \alpha_{t} y_{i} h_{t} (x_{i} )} \right\}}}{{N_{t} }}$$

where ${N}_{t}$ is a normalization constant and ${\sum }_{i=1}^{n}{w}_{i}^{t+1}=1$

Step 7: The final classifier is given by

21 $$H(x) = {\text{sgn}} \left[ {\sum\nolimits_{t = 1}^{T} {\alpha_{t} h_{t} (x)} } \right]$$

The LOSOCV approach is given by the expression: $1\left/ 2n\right.=\sum_{t=1}^{n}\left|{f}_{i}\left({x}_{i}\right)-{l}_{i}\right|$, where *n* represents the total trained data.

### Facial expression datasets

The algorithm was trained and tested on five popular datasets: Bosphorus, BU-3DFE, MMI, CK + , and BP4D-Spontaneous, and executed on a (4 CPUs), approximately 2.2 GHz processor with a memory capacity of 8192 MB RAM.

## Results and discussion

### Experiments on databases

**Fig. 6:**
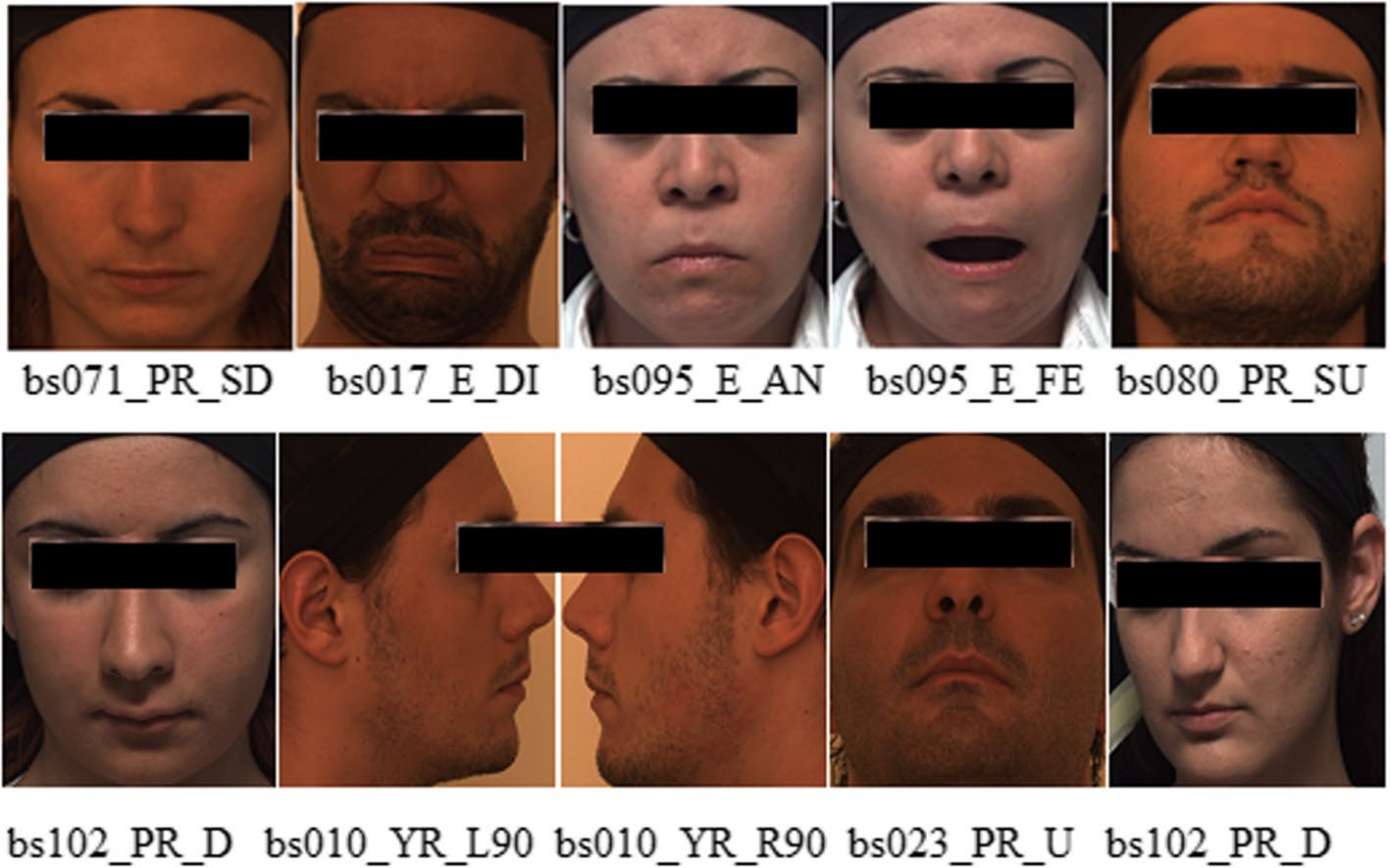
Sample Bosphorus datasets

Bosphorus contains 4666 images of 105 subjects [26] comprising 60 men and 5 women, with the majority being Caucasian; 27 of whom were professional actors, in various poses, expressions, and occlusion conditions. In addition to the 6 basic emotional expressions, various systematic head poses (13 yaw and pitch rotations) were present. The texture images have a resolution of 1600 × 1200 pixels, whereas the 3D faces comprise approximately 35,000 vertices [27]. Figure 6 presents sample datasets from Bosphorous. Occlusion images were discarded because they were not the focus of this study. The datasets used comprised 6 poses and 7 expressions. The images were partitioned into training and testing sets using the conventional LOSOCV approach. One specimen from each of the 6 groups of expressions was used as a test dataset during each training run, whereas the rest of the samples were used as a testing set. Table 1 summarizes the FER in Bosphorus.

**Table 1:** FER in Bosphorus database

Pose	Expression	Average recognition (%)	Expressions	Average recognition (%)
10^0^ Yaw	Neutral	100	Happiness	99.2
20^0^ Yaw	Neutral	99.8	Sadness	98.0
30^0^ Yaw	Neutral	99.2	Disgust	98.4
L45^0^ Yaw	Neutral	97.3	Angry	99.4
R45^0^ Yaw	Neutral	97.8	Fear	99.6
L90^0^ Yaw	Neutral	63.2	Surprise	99.0
R90^0^ Yaw	Neutral	78.2	Overall average	98.9
PR	Neutral	99.7		
CR	Neutral	98.9		

Average recognition accuracy = 92.7%

**Fig. 7:**
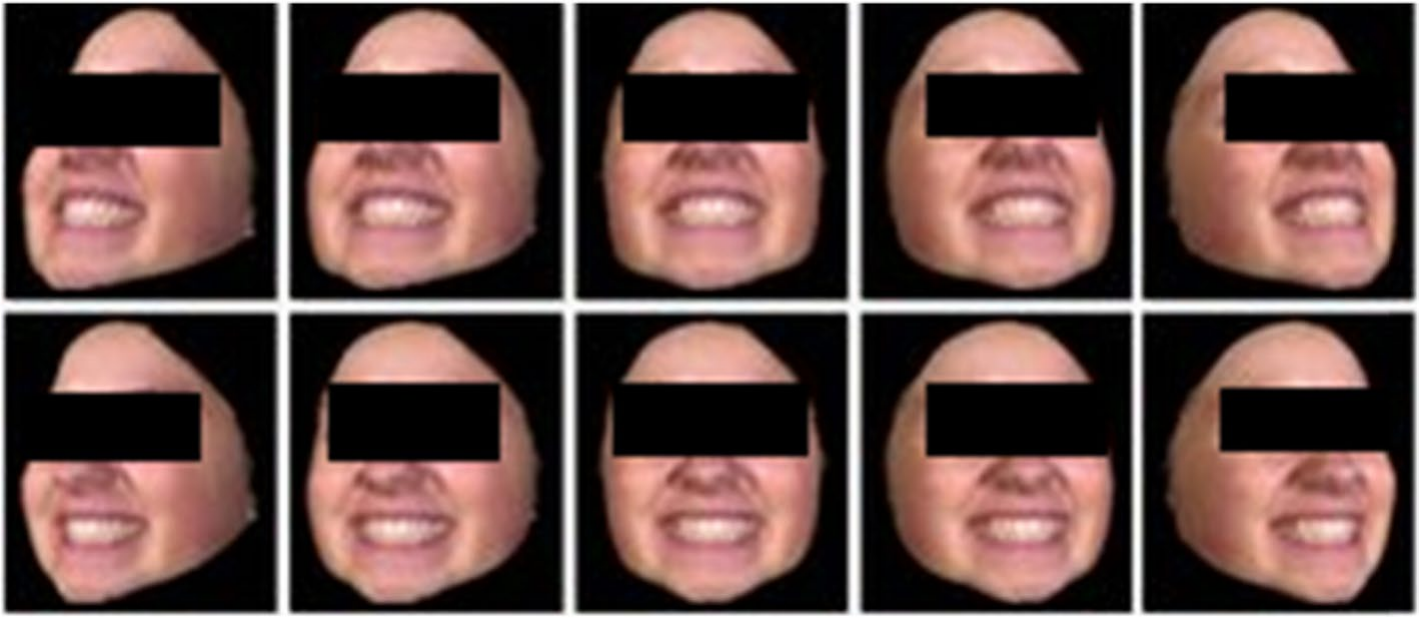
Sample BU3DFE datasets

The BU-3DFE database was created at Binghamton University [28]. There were 100 respondents, ranging in age from 18 to 70 years old. Whites, Blacks, East Asians, Middle East Asians, Indians, and Hispanics are among the ethnic groups. Each participant displayed 7 expressions at 4 intensity levels, including neutral, and 6 archetypal facial expressions. Figure 7 shows sample datasets in the database. The images were separated into training and testing sets using the same LOSOCV method as that used for the Bosphorus datasets, and the average recognition accuracy was 94.56%.

**Fig. 8:**
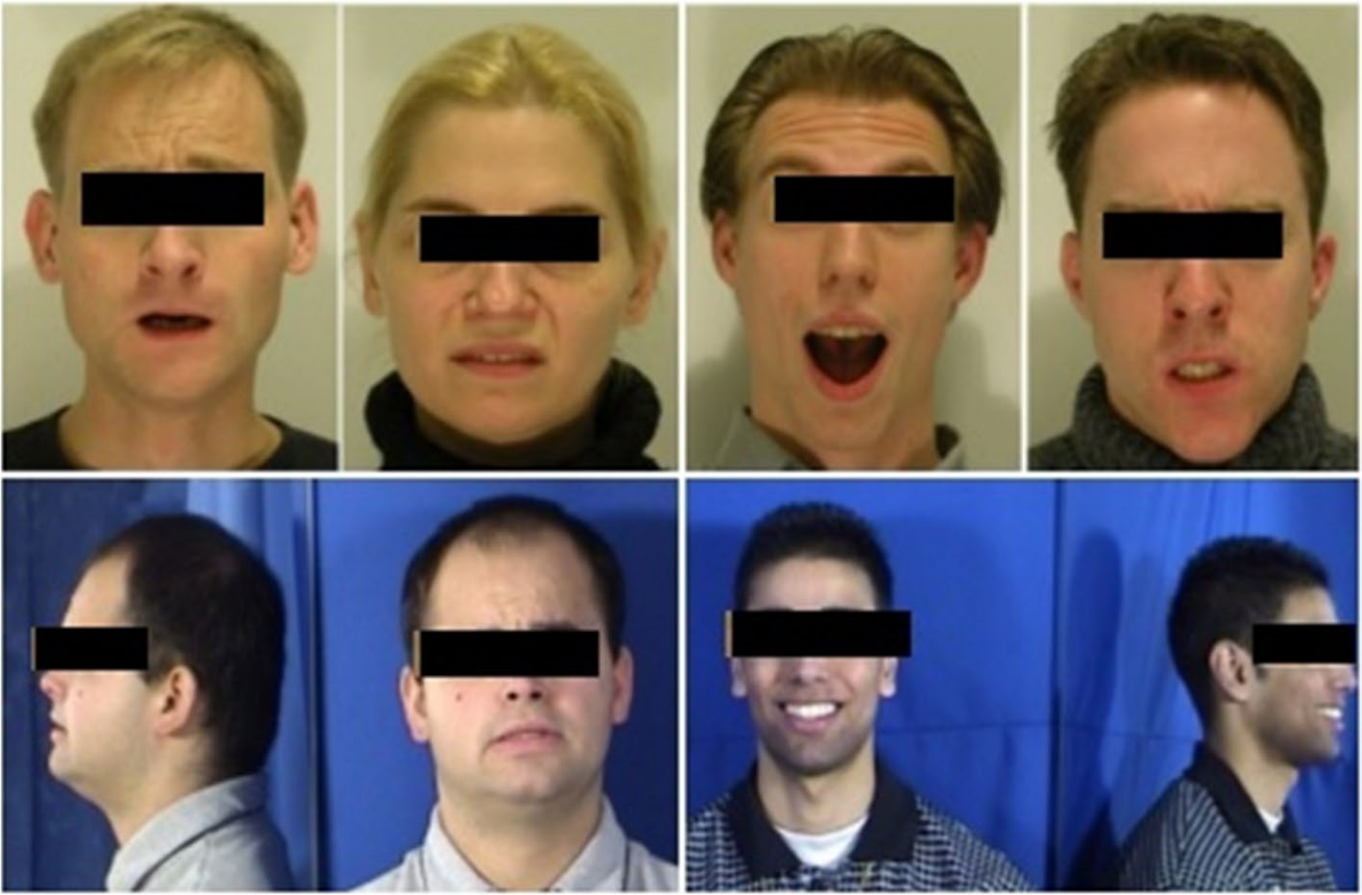
Sample MMI datasets

The MMI database comprises over 2900 high-resolution videos submitted by more than 20 students and research staff members, of which 44% are female, ranging in age from 19 to 62 years old. Seventy-five subjects were included in total, and Fig. 8 shows samples. The datasets are partitioned into training and testing sets using the LOSOCV technique. One sample from each of the 7 types of expressions was used as the test dataset during each training run. The remaining samples were used as training sets. For each training cycle, the samples were repeated with new test samples. The expressions included anger, disgust, fear, happiness, neutral, sadness, and surprise. The average recognition accuracy is 97.2%.

**Fig. 9:**
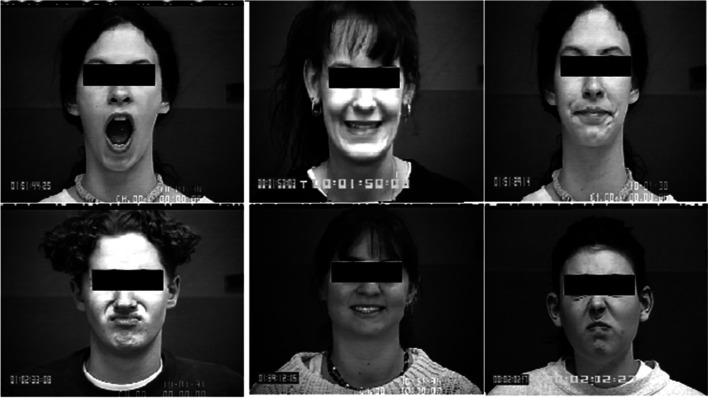
Sample images in CK + database

The CK + database is a version of the 210 adult CK database. Participants were 18 to 50 years old, with 69% female, 81% Euro-American, 13% Afro-American, and 6% from other ethnic groups. The expressions included anger, contempt, disgust, fear, happiness, sadness, and surprise. Figure 9 presents sample datasets. A tenfold cross-validation procedure was used to partition the datasets into training and testing sets. The average recognition accuracy is 99.48%.

**Fig. 10:**
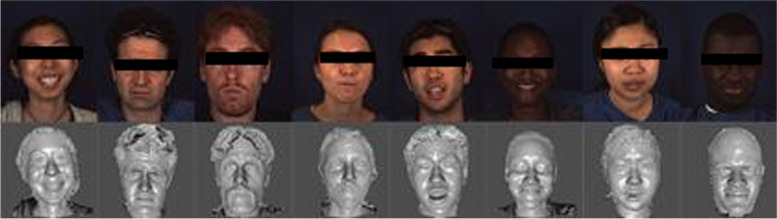
Sample BP4D-Spontaneous datasets

Finally, the BP4D-Spontaneous dataset is a 3D video collection of spontaneous facial expressions from young individuals. The database comprises 41 subjects (23 women and 18 men) ranging in age from 18 to 29 years old, including 11 Asians, 6 African-Americans, 4 Hispanics, and 20 Euro-Americans. Figure 10 shows sample images. We extracted expressions of anger, disgust, fear, pain, happiness, sadness, and surprise. The datasets were partitioned into training and testing sets using tenfold cross-validation. The average recognition accuracy is 97.2%.

**Fig. 11:**
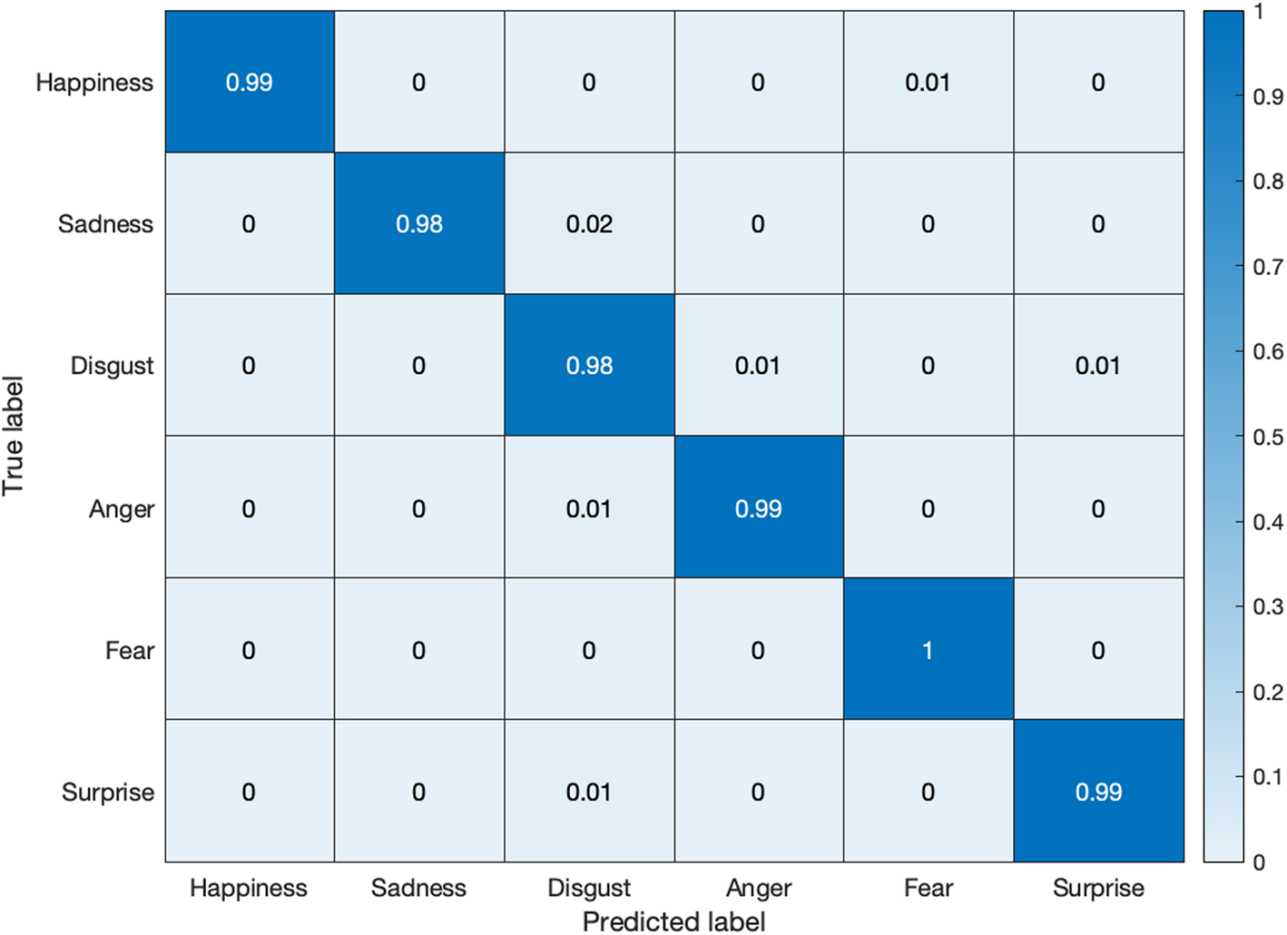
Confusion matrix of facial expressions in Bosphorus

**Fig. 12:**
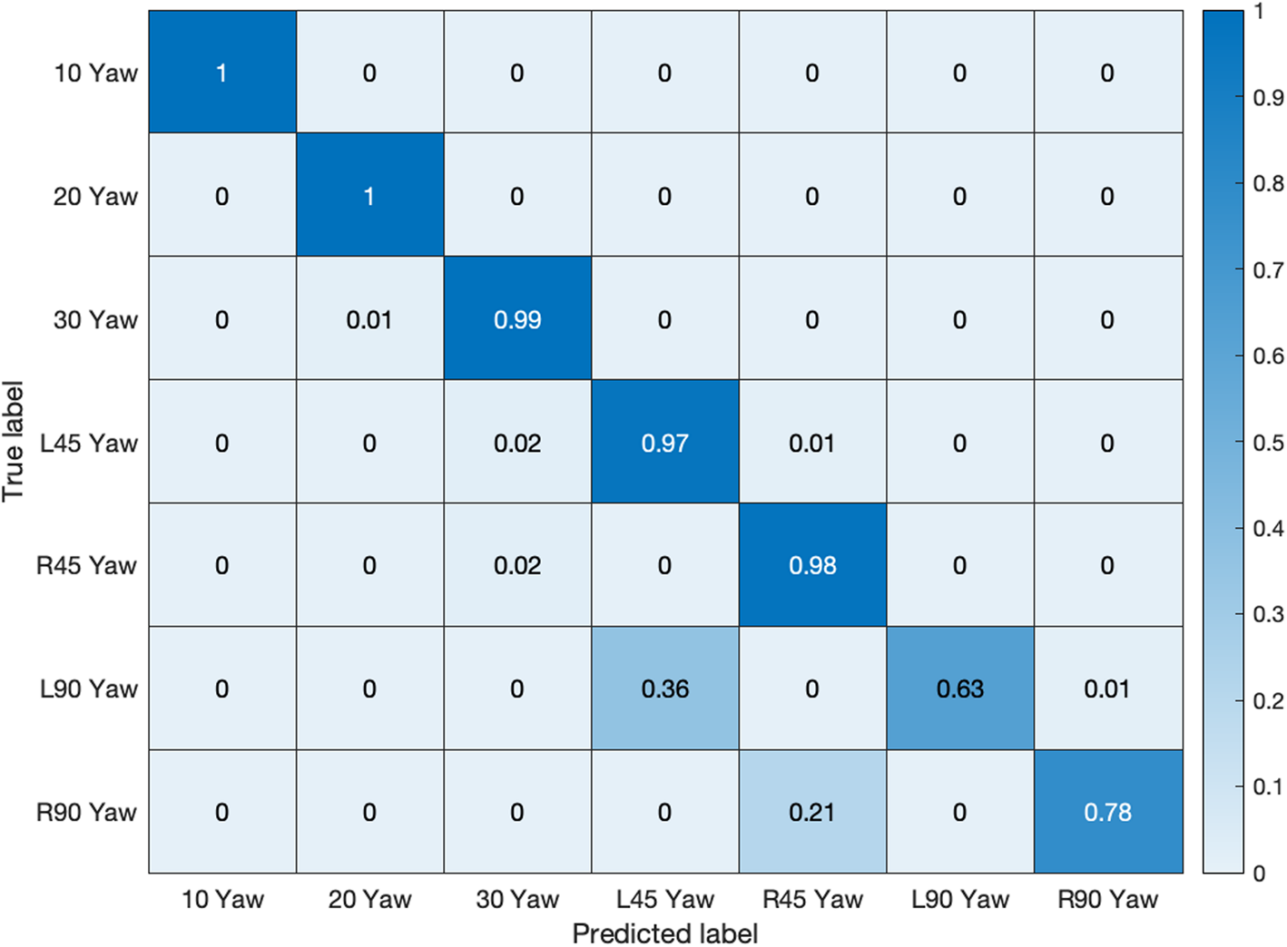
Confusion matrix of pose prediction in Bosphorus

**Fig. 13:**
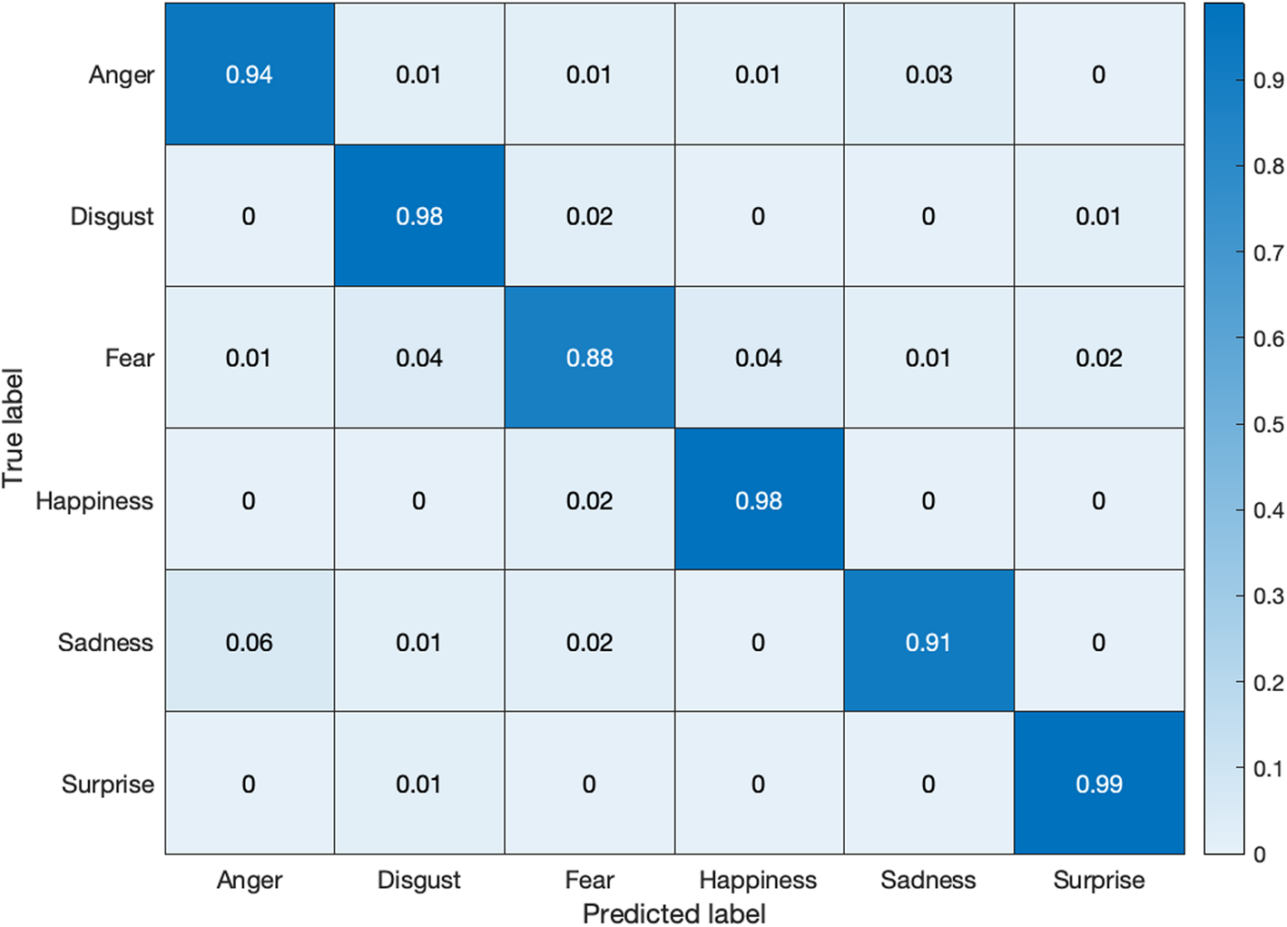
Confusion matrix of facial expressions in BU3DFE database

**Fig. 14:**
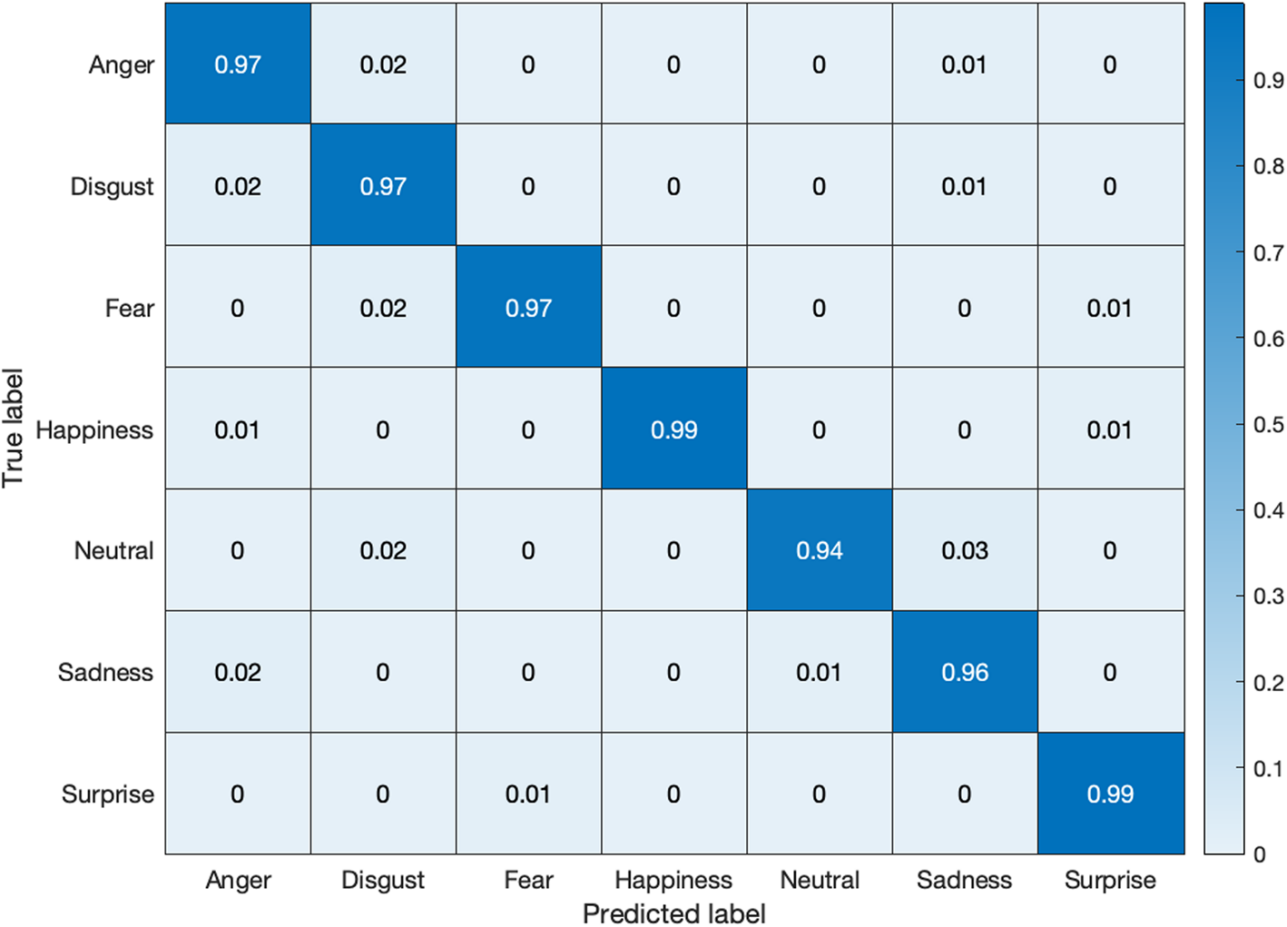
Confusion matrix of facial expressions in MMI database

**Fig. 15:**
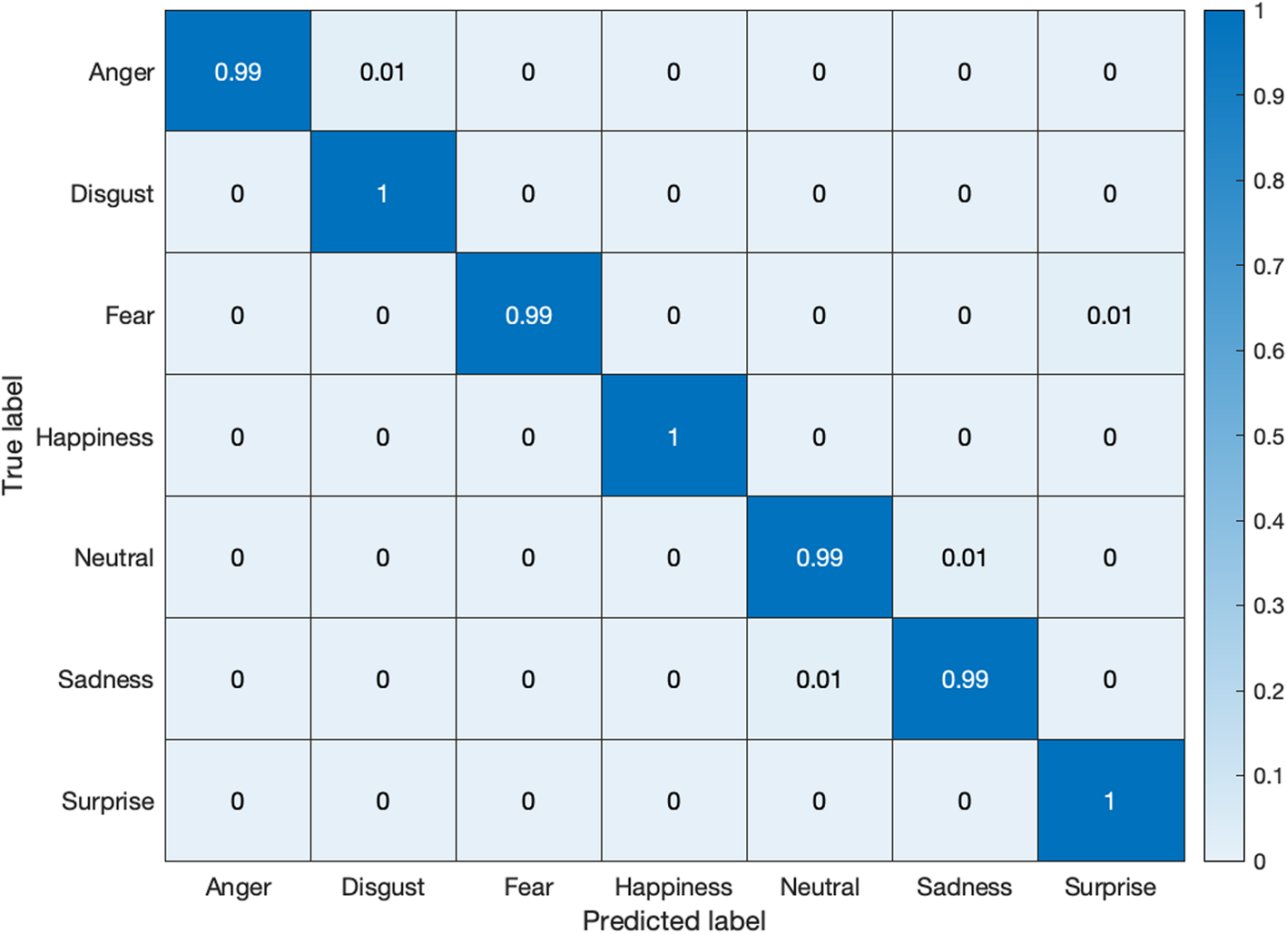
Confusion matrix of facial expressions in CK + database

**Fig. 16:**
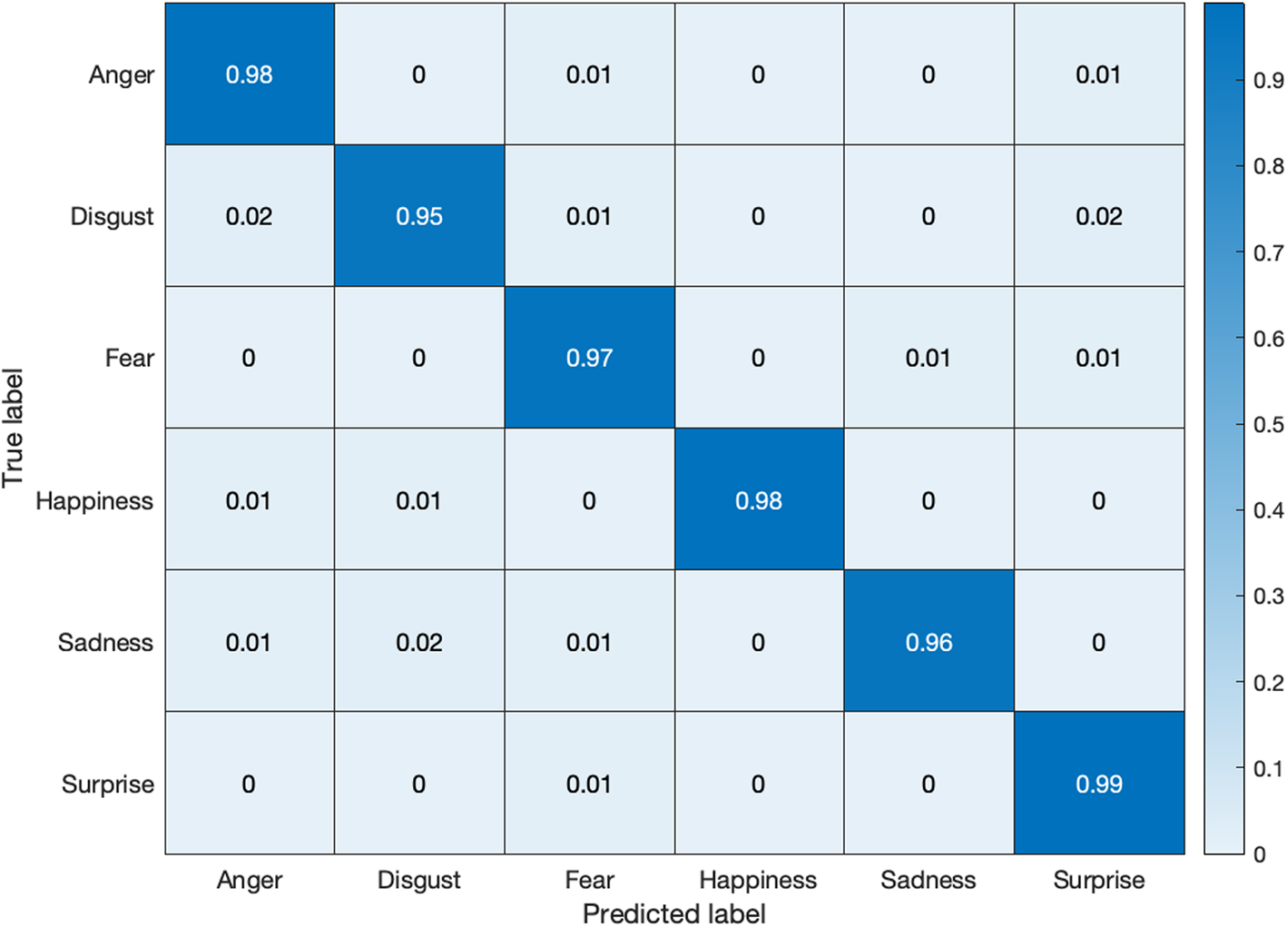
Confusion matrix of facial expressions in BP4D-Spontaneous datasets

Figures 11 and 12 exhibit the respective confusion matrices for facial expressions and pose predictions in the Bosphorus database. Figures 13, 14, 15, and 16 show the rest of the confusion matrices for FERs in BU3DFE, MMI, CK + , and BP4D-Spontaneous, respectively.

### Comparison of methods

**Fig. 17:**
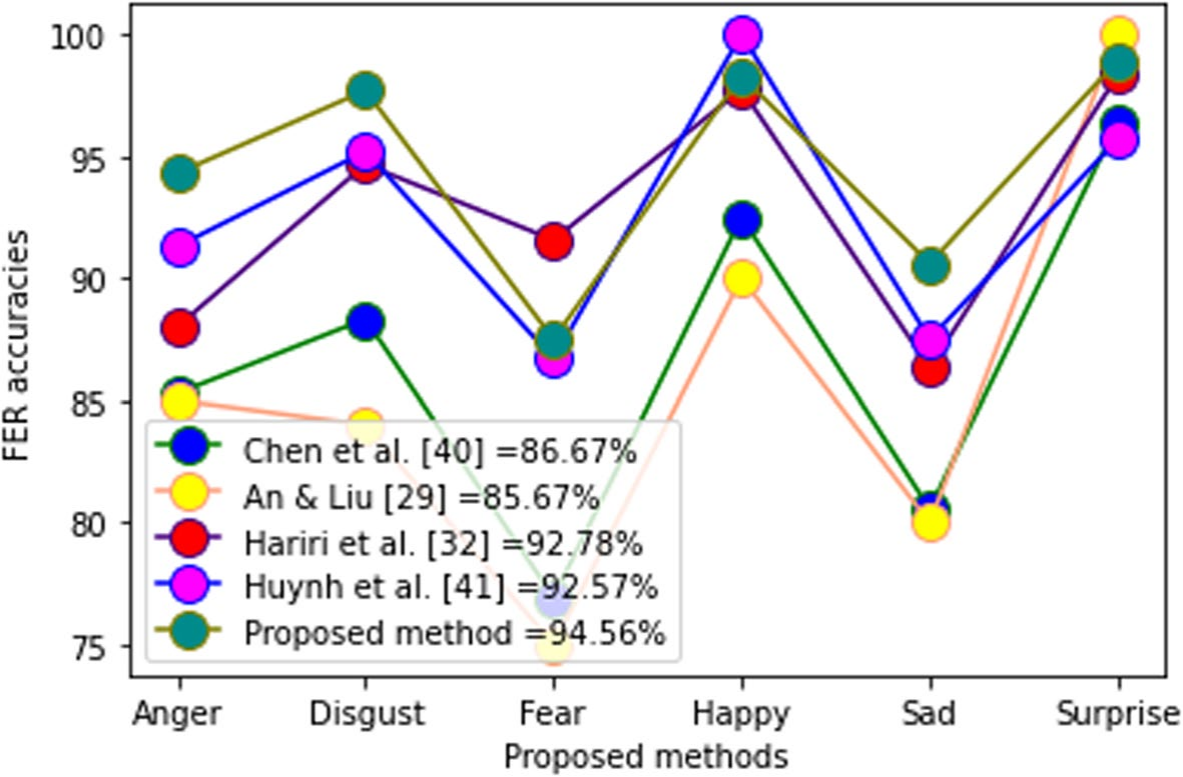
Performance of 6 FER prototypes in BU3DFE database

**Fig. 18:**
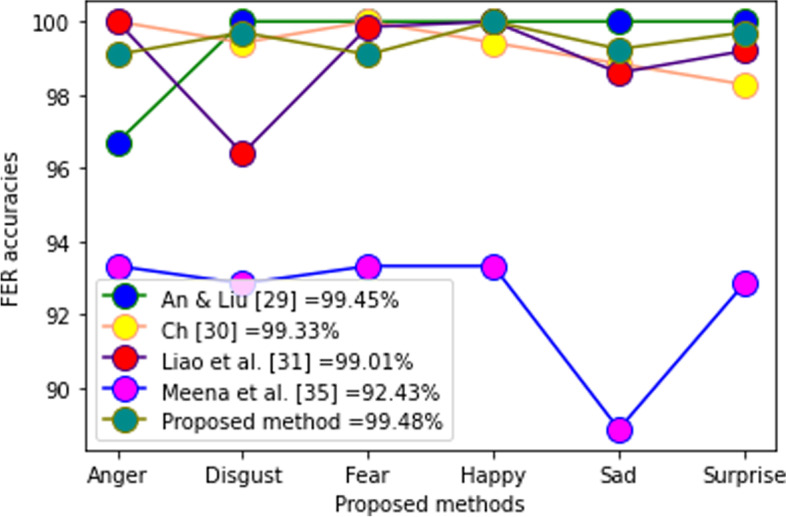
Performance of 6 FER prototype in CK + database

**Fig. 19:**
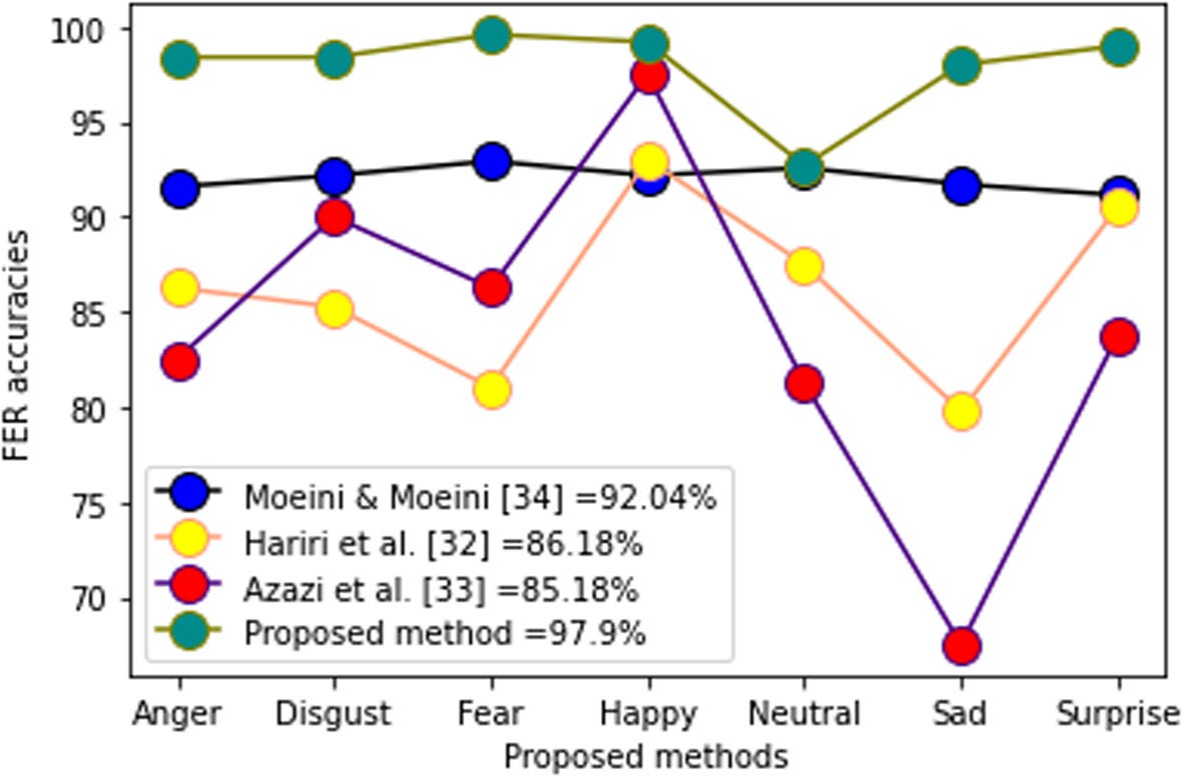
Performance of 7 FER prototypes in Bosphorus database

In Table 2, the proposed method was compared to some recent techniques. These results clearly demonstrated that the proposed method is promising. Figures 17, 18, and 19 show the performance of each of the 7 facial expressions. In the BU3DFE database, many authors failed to report the performance of neutral expressions; thus, the comparison was performed using the other 6. The performance shown in Fig. 17 was encouraging. Figure 18 shows the performance of the CK + database. Although the result, as shown in Fig. 18, depicts fierce rivalry between three current methods [29–31], the overall average recognition shows that the proposed technique is promising. In the Bosphorus database, the proposed method outperformed the most recent methods (Fig. 19). A comparison of the performances of the individual FER prototypes in the MMI and BP4D-Spontaneous databases could not be executed because there were no reported data for comparison at the time of compilation. Statistical analysis using ANOVA shows the following performance results:

**Table 2:** Comparison of results on different methods

Method	Database	Recognition (%)	Ref
Twin support vector machines classifier	MMI	$92.56\pm 3.02$	[32]
DBM-DACNN with entropy loss	MMI	79.25	[33]
Deep learning neural network-regression	CK +	97.27	[30]
Deep learning + random forest	CK +	99.00	[31]
Twin support vector machines classifier	CK +	$93.42\pm 3.25$	[32]
DBM-DACNN with entropy loss	CK +	96.46	[33]
Geotopo +	BP4D-Spontaneous	88.56	[34]
Two-phase weighted collaborative representation classification	BP4D-Spontaneous	100	[35]
Fine-grained matching of 3D keypoint descriptors	Bosphorus	98.90	[21]
Kernel methods on Riemannian manifold	Bosphorus	86.70	[36]
SVM with EPE	Bosphorus	84.00	[37]
Two-phase weighted collaborative representation classification	Bosphorus	98.90	[35]
Kernel methods on Riemannian manifold	BU-3DFE	92.62	[36]
SVM with EPE	BU-3DFE	85.81	[37]
Manifold CNN	BU-3DFE	86.67	[38]
CNN model	BU-3DFE	92.57	[39]
Proposed method	MMI	97.20	This study
Proposed method	CK +	98.20	This study
Proposed method	BP4D-Spontaneous	97.20	This study
Proposed method	Bosphorus	98.90	This study
Proposed method	BU-3DFE	93.50	This study

In the Bosphorus database, an analysis of variances demonstrated statistically significant differences between the proposed technique and the following: Hariri et al. [36] (*p* = 0.001), Azazi et al. [37] (*p* = 0.000), and Moeini A and Moeini H [40] (*p* = 0.013). In addition, the outcome is the same as in the BU3DFE: the variance analysis shows that a statistically significant difference (*p* < 0.05) exists between the proposed method and all other methods. However, in the CK + FER database, the statistical analysis shows that, except ref. [41], where a statistically significant difference (*p* < 0.05) exists, the remaining datasets show no statistically significant differences (*p* > 0.05). The proposed method compared to yields from An and Liu [29] (*p* = 0.847), Ch [30] (*p* = 0.909), and Liao et al. [31] (*p* = 0.991). Although the analysis appears to reveal a balanced performance between the proposed methodology and the last three techniques, the average recognition accuracy of the proposed method against any of them, as shown in Fig. 18, indicates that the proposed method is superior.

## Conclusions

This study improves the FER performance in higher poses. 2D pose conversion schemes have been established to handle pose-invariant FER problems successfully, within a small-scale pose variation. However, they often flop for large-scale, in-depth face variations because of the disjointedness of the image. Human face geometry is ellipsoidal; therefore, the feature points are robustly tracked from one frame to next using an ellipsoidal model. We use the Gabor feature extraction technique for the salient visible features, mostly around the cheeks, eyes, mouth, and nose ridges. The Gabor feature extraction algorithm is useful for this study because it is selective toward orientation, localization, and frequency. We then used an ensemble classification technique, which combines SVM and AdaBoost, for feature selection and classification. The proposed technique outperforms the most recent and popular methods. In the future, we intend to investigate this problem using other feature extraction methods such as LBP and LBP + HOG.

## Data Availability

All data used for this data are publicly available on request from the original authors.

## Abbreviations

FER: Facial expression recognition
SVM: Saturated vector machine
LBP: Local binary patterns
HOG: Histogram of gradients
PCA: Principal component analysis
KNN: K-nearest neighbor
SMOTE: Synthetic minority oversampling technique
2D: Two-dimensional
3D: Three-dimensional
LOSOCV: Leave-one-subject-out cross validation
